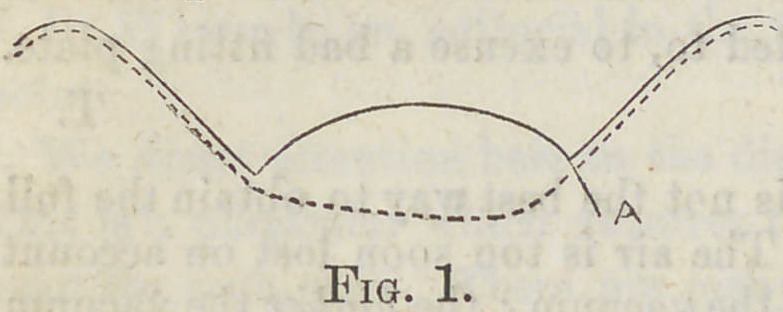# Atmospheric Pressure Plates

**Published:** 1862-03

**Authors:** 


### Editorial.

---

### ATMOSPHERIC PRESSURE PLATES.

The following remarks upon atmospheric pressure plates, by Dr. J. D. White, in an editorial in the Cosmos, are good and to the point.

We direct attention here to the diameter and depth of chambers. This is a particular which requires the exercise of a correct judgment for each case. There are many cases in which the depth of chamber specified by the Doctor is not really required; for instance, when there is but little tissue between the bone and mucous membrane—when the surface of the arch is firm and smooth; in such cases we have been accustomed to make the chambers larger in diameter and of less depth than when the tissues are thick and soft.

It is possible that the Doctor's plan is better than ours, even here; we adopted the plan, perhaps, more from analogy than from actual experiment; we will, however, put out our *feelers* a little farther in this direction.

In regard to another particular the Doctor is certainly correct, and that is the sharpness or definiteness of the border of the chamber. This is a point that has been altogether too much overlooked in practice; the more definite and sharp the borders of the chamber, the more perfectly will the air be excluded, after it is exhausted. In this consists the advantage of the Cleveland chamber. The efficiency of the chamber consists in the perfect exclusion of the air, rather than in its capacity; at least it is commonly regarded so. As a general rule, we think, the distance from the center of the ridge to the margin of the chamber should be the same at every point, and the distance from the palatine edge of the plate to the posterior border of the chamber should be the same as that specified above.

In the use of chambers there should be much more discrimination than now obtains in common practice. There are many cases that operate just as efficiently without chambers; and again different cases will require different sizes and different forms of chambers. They are always objectionable, so far as they fill the arch ; the tongue and other parts of the mouth will accommodate themselves very much to the new order of things ; nevertheless we think it desirable to avoid this change as far as possible. Then again, there is in some instances an intolerance of the mucous membrane to these changes, or to the vacuum produced in them. The highest skill will certainly endeavor to avoid the use of them whenever possible. They are often resorted to, to excuse a bad fitting plate.

T.

A shallow and broad cavity is not the best way to obtain the full value of atmospheric pressure. The air is too soon lost on account of the *shortness*, so to speak, of the vacuum ; the *higher* the vacuum the longer it will require to be filled by air, and the more readily will the patient be enabled to exhaust it by suction. We may say that the patient has more leverage on an operation, and therefore more power over it. The variation from the rule we will leave to the judgment of each operator as to whether the cavity may be shallow, wide, or narrow ; but to depend upon the alveolar border for atmospheric pressure, a part which is changing as long as a patient lives, is simply absurd in itself ; the stationary point, the roof of the mouth, is the place to work from. We hope we will be excused for making this article a little desultory. A distinguished judge called to see us about a month since, for whom we had placed in the mouth an entire set of artificial teeth two years ago. He could exhaust the air from the cavity, and the job would be fixed firmly to the roof of the mouth ; but on attempting to chew, or in speaking for a length of time, the teeth would drop; the gums had changed since the plate had been made. He suggested the propriety of reconstructing it so as to obtain a better fit, which would, in all probability, overcome the difficulty complained of. As we regarded the case a failure, we concluded to try an experiment with it, as we could not make it less useless. We held the set of teeth in the palm of the hand, and battered the cavity deeper with a small riveting hammer, and placed the job in the mouth. "Why," he remarked, "what have you done to it ? It never felt so firm before! I have no trouble in exhausting the air at all !" The teeth are now useful in mastication. We have *stretched* a good many cavities since then, successfully.

We will cite another case, of which a drawing will be found below. Five or six years since we made a plate with three or four teeth, bicuspids, adapted the plate well ; the pressure was complete, but the case useless for chewing. It was laid aside for a time. As the front teeth of the patient were wearing away very rapidly, he called to see us again, about one year ago, hoping to get something made to chew on, as the front teeth had become short and tender, and one or two more back teeth had been lost. As we had an assistant who was one of the very best and most ingenious operators in artificial work, we placed the patient under his hands ; a *vulcanite* plate was proposed, because the *metal* plate had failed. The job was made up, the fit was complete, but failed as well as the former one. It was let alone until recently, when we had a silver plate struck up with a chamber constructed as represented in Fig. 1, but a little broader than was intended, or is required. Still it answers every purpose for chewing ; the pressure can be graded to suit the wants of the patient. The black line represents the plate, the dotted line the roof of the mouth. We always swedge up the plate so that the margin of the chamber at A shall be as sharp as possible, and fill in the upper part of the plate so that the sharp margin can be reduced at will without cutting through the plate at that point, in case it is too severe on the mouth. We also double the plate back of the chamber, so that if in time it impinges too hard on the roof of the mouth, it can be filed away. A plate which can not be altered from time to time is in a manner useless to a patient. We take great care now to spring the chamber like an arch directly from the gum, as we can get a closer fit than the old way. We also place the cavity on the roof of the mouth, and as free as possible from contact with the corrugations frequently met with on the anterior part of it. A plate constructed with a cavity or chamber is a *suction plate*, and nothing else ; and it is the best way to avail ourselves of the full force and advantages of atmospheric pressure for dental purposes.

## Figures and Tables

**Fig. 1. f1:**